# Soluble P-selectin and correlation with Prothrombin Fragment 1 + 2 in myeloid malignancies in Cipto Mangunkusumo general hospital

**DOI:** 10.1186/s12959-021-00307-5

**Published:** 2021-07-30

**Authors:** Lugyanti Sukrisman

**Affiliations:** Division of Hematology and Medical Oncology, Department of Internal Medicine, Faculty of Medicine, Universitas Indonesia - Cipto Mangunkusumo General Hospital, Jakarta, Indonesia

**Keywords:** sP-selectin, F1 + 2, AML, CML

## Abstract

**Background:**

Myeloid cells express microparticles that could increase the expression of adhesion molecules including P-selectin. We aimed to evaluate the level of soluble P-selectin (sP-selectin) and prothrombin fragment 1 + 2 (F1 + 2), and to determine correlation of sP-selectin with leukocyte count and F1 + 2 levels in acute myeloid leukemia (AML) and chronic myeloid leukemia (CML) patients.

**Methods:**

Patients with newly diagnosed AML (*n* = 25), CML (*n* = 13), and controls (*n* = 17) were recruited in this study. The diagnosis of AML and CML is based on 2001 WHO and/or FAB criteria. Levels of sP-selectin and F1 + 2 were determined using enzyme-linked immunosorbent assay kits (Behring ELISA Processor-III® and Behring Enzygnost F1 + 2).

**Results:**

sP-selectin was significantly elevated in CML patients compared to AML patients (*p* = 0.001). Levels of F1 + 2 in AML and CML patients were significantly increased in comparison to controls (*p* < 0.001 and *p* = 0.043). Levels of sP-selectin were significantly correlated to leukocyte count (*r* = 0.437; *p* = 0.029) and F1 + 2 (*r* = 0.436; *p* = 0.029) in AML patients.

**Conclusions:**

AML and CML patients had an increased tendency to thrombosis. While CML patients had higher platelet and/or endothelial activation, hypercoagulable state are more pronounced in AML patients.

## Background

Malignancies are associated with the development of thromboembolic event, approximately up to six-fold higher than the general population. The pathogenesis of cancer-associated thrombosis (CAT) is multifactorial and complex, mainly related to components of Virchow’s triad [1, 2]. Cancer cells may alter hemostasis by secreting procoagulant factors and interacting with endothelial cells to trigger a coagulation cascade [3].

Patients with hematological malignancy also carry an increased risk of cancer-associated thrombosis. A recent meta-analysis reported that venous thromboembolism (VTE) events were highest in CML patients among other leukemia subtypes [4]. Circulating microparticles and adhesion molecules play an important role in the mechanism of thrombus formation in hematologic malignancies [5].

P-selectin is expressed on activated platelets and endothelium. Interaction with its ligand, P-selectin glycoprotein ligand 1 (PSGL-1), on leukocytes mediates adherence of leukocytes to endothelium and activated platelets and shedding of P-selectin into the circulation as soluble P-selectin (sP-selectin). Soluble P-selectin, in dimeric or multimeric form, may play a role in thrombogenesis by forming a stable leukocyte-platelet aggregates [6–8].

Prothrombin fragment 1 + 2, thrombin-antithrombin complex (TAT), and soluble fibrin have been reported to reflect thrombin formation in early phase of thrombosis, whereas D-dimer reflects fibrin degradation products in secondary fibrinolysis [9–11]. Ota et al. found that levels of F1 + 2 in plasma were significantly correlated to TAT, soluble fibrin, and D-dimer. They found that with a cutoff value of F1 + 2 for diagnosis of thrombosis of 300 pmol/L, the sensitivity and specificity were 86.2 and 80.6% respectively [9]. Thus, F1 + 2 is considered as a useful parameter for a hypercoagulable state and for the diagnosis of all types of thrombosis.

The aim of this study was to investigate levels of sP-selectin in myeloid malignancies (AML and CML) patients, and to evaluate its correlation with activation coagulation (F1 + 2) and leukocyte count that specific for hematologic malignancy.

## Subjects and method

This was a cross-sectional study conducted at Hematology - Medical Oncology Division in Cipto Mangunkusumo General Hospital, Jakarta, Indonesia, from January to June 2012. Subjects were newly diagnosed AML and CML based on WHO and/or French-American British (FAB) criteria, aged 18 years or older. We also collected samples from healthy individuals (controls) who were free of any diseases, as confirmed by annual medical checkup, and did not take any anticoagulant and/or antiplatelet medication. Patients were excluded from the study in case of pregnancy, severe infection, immobilization for at least 3 days, during intensive chemotherapy, or refused to participate in the study. The study protocol was approved by the local Ethics Committee from Faculty of Medicine, Universitas Indonesia. All subjects signed the informed consent forms before the study.

Venous blood sample was drawn from each subject before starting chemotherapy and divided into an EDTA vacutainer for complete blood count and sP-selectin measurements, and into a 3.8% sodium citrate (9 parts of whole blood and 1 part of sodium citrate at 0.129 mmol/L) vacutainer for the determination of F1 + 2. Platelet-poor plasma was obtained by blood centrifugation, then aliquoted and stored at − 20 °C until testing.

Hemoglobin (Hb), platelet counts, and leukocyte counts were determined using an automated blood analyzer (ABX Micros 60® analyzer). Commercial enzyme-linked immunosorbent assay/ELISA kits were used to measure plasma levels sP-selectin (Behring ELISA Processor-III®, Dade Behring) and F1 + 2 (Enzygnost F1 + 2, Dade Behring) according to manufacturer’s instructions.

### Statistical analysis

The data were not normally distributed, hence expressed as median and interquartile range (IQR). Statistical significance between two groups were determined using Mann-Whitney U test for nonparametric variables. The correlation between two variables was evaluated by Spearman’s correlation analysis. A *p*-value < 0.05 indicates statistically significant difference. All statistical analysis was performed with SPSS version 24 for Mac.

## Results

A total of 55 subjects were enrolled in the study. The study population comprised of 17 healthy subjects (controls), 25 AML patients, and 13 CML patients. In control group, the median age was 40 years (IQR 22 years) and 70.59% of subjects were males. In AML group, the median age at diagnosis was 32 years (IQR 17 years) and 60% of subjects were males. In CML group, the median age at diagnosis was 47 years (IQR 32 years) and 53.85% of subjects were males. Hematological characteristics of the subjects are shown in Table 1. AML and CML groups had lower hemoglobin (Hb) and marked increased of leukocyte and blast count as compared to controls. Platelet count was markedly reduced in AML group.

**Table 1:** Characteristics of the subjects

	Control (*n* = 17)	AML (*n* = 25)	CML (*n* = 13)
Age (years), median (IQR)	40 (22)	32 (17)	47 (32)
Sex			
Male, n (%)	12 (70.59)	15 (60)	7 (53.85)
Female, n (%)	5 (29.41)	10 (40)	6 (46.15)
Hb (g/dL), median (IQR)	13.9 (2.3)	8.1 (2.2)^*^	8.6 (3.95)^*^
Platelet count (×10^3^/mm^3^), median (IQR)	308 (88)	67 (14.12)^*^	417 (419)^§^
Leukocyte (×10^3^/mm^3^), median (IQR)	7.8 (2.3)	52.29 (93.76)^*^	60.3 (16.9)^*^
Peripheral blast count (%), median (IQR)	0 (0)	44 (43.5)^*^	3 (15.5)^*§^

*IQR* interquartile range

^*^ *p* < 0.001 vs. control; ^§^ *p* < 0.001 vs. AML

**Fig. 1:**
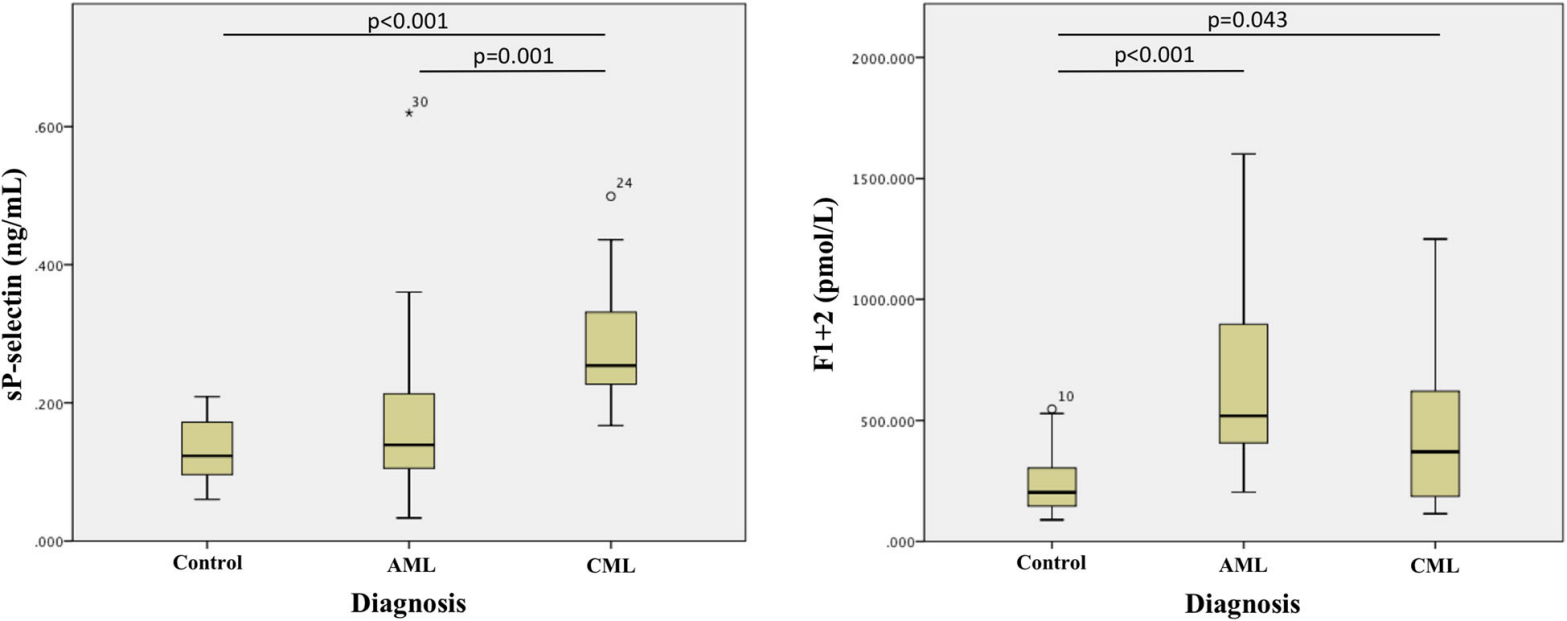
Plasma levels of sP-selectin and F1 + 2. The box shows 25th percentile, median, and 75th percentile. A *p*-value < 0.05 indicates statistically significance

As shown in Table 2 and Fig. 1, AML patients had increased levels of sP-selectin and F1 + 2 compared to controls, but only F1 + 2 reaching statistically significant (median 519.03 pmol/L; *p* < 0.001 vs. controls). CML patients had significantly increased levels of both sP-selectin (median 0.25 ng/mL) and F1 + 2 (median 370.16 pmol/L) compared to controls (*p* < 0.001 and *p* = 0.043 respectively). While sP-selectin levels were significantly higher in CML than in AML patients (median 0.25 ng/mL vs. 0.14 ng/mL, *p* = 0.001), F1 + 2 levels were higher in AML than in CML patients, despite being not statistically significant.

**Table 2:** Plasma levels of sP-selectin and F1 + 2

	Control (*n* = 17)	AML (*n* = 25)	CML (*n* = 13)
sP-selectin (ng/mL), median (IQR)	0.12 (0.09)	0.14 (0.12)	0.25 (0.14)^Δϑ^
F1 + 2 (pmol/L), median (IQR)	201.99 (173.64)	519.03 (563.2)^Δ^	370.16 (477.55)^Δ^

*IQR* interquartile range, F1 + 2 prothrombin fragment 1 + 2

^Δ^ Significant difference compared to control at *p* < 0.05

^ϑ^ Significant difference compared to AML at *p* < 0.05

The correlation analysis according to disease type showed that sP-selectin was significantly correlated to either leukocyte count (*r* = 0.437; *p* = 0.029) and F1 + 2 (*r* = 0.436; *p* = 0.029) in AML patients only. There was no significant correlation among leukocyte count, sP-selectin, and F1 + 2 in controls and CML patients (Table 3).

**Table 3:** sP-selectin correlation with Leukocyte count and F1 + 2

Variables	Control (***n*** = 17)		AML (***n*** = 25)		CML (***n*** = 13)	
	Correlation coefficient^a^ (r)	*p*	Correlation coefficient^a^ (r)	*p*	Correlation coefficient^a^ (r)	*p*
Leukocyte count vs sP-selectin	0.043	0.87	0.437	**0.029**	0.44	0.133
sP-selectin vs F1 + 2	−0.088	0.736	0.436	**0.029**	−0.253	0.405
Leukocyte count vs F1 + 2	−0.115	0.66	0.12	0.568	0.049	0.873

*AML* acute myeloid leukemia, *CML* chronic myeloid leukemia

^a^ Spearman correlation

*p* < 0.05 is considered statistically significant

## Discussion

This present study investigated the plasma levels of sP-selectin, a marker of platelet and endothelial activation, and F1 + 2, a marker of early phase of thrombosis, in 17 controls and 38 patients with AML and CML.

P-selectin is an adhesion molecule stored in Weibel-Palade bodies of endothelial cells and alpha-granules of platelets. It is the largest of the known selectins, along with E-selectin (expressed by endothelial cells) and L-selectin (expressed by leukocytes) [8, 12]. Several studies reported that acute leukemia are related to higher platelet activation. Mechanisms leading such activation are: (1) leukemic cells cause endothelial cells damage while invading bone marrow, body tissues, and organs; (2) leukemic cells release procoagulant molecules; (3) leukemic cells express adhesion molecules; (4) chemotherapeutic agents cause endothelium damage; (5) leukemic cells release higher levels of cytokine and chemokines [13]. Cytokines released by blast cells can induce the expression of P-selectin [8].

Upon activation, P-selectin is mobilized to the external membrane to mediate leukocyte adherence [8, 12]. Role of P-selectin in coagulation cascade can be explained by two ways. First, P-selectin may support platelet-platelet interaction. Secondly, P-selectin on platelet surface promotes leukocyte recruitment and facilitates binding of tissue factor-bearing microparticles (TF-MP), derived from normal cells or cancer cells, to leukocytes via PSGL-1 [8, 14, 15]. Additionally, adhesion of leukocytes to P-selectin via PSGL-1 induces shedding of P-selectin into the circulation. Increased concentrations of soluble form of P-selectin (sP-selectin) from activated platelets or activated endothelial cells are associated with VTE events in cancer patients [16, 17]. A possible mechanism is that sP-selectin can activate leukocytes via PSGL-1 to generate TF-MP which contribute to pro-coagulant state [11, 18].

In the present study, plasma levels of sP-selectin were increased significantly in CML patients in relation to AML patients and controls. This findings were supported by Cella et al. who demonstrate a significant increased levels of sP-selectin in myeloproliferative neoplasms (MPN) patients compared to controls [12]. Furthermore, this might be related to higher platelet counts in CML patients than in AML patients, as seen in our patients (median 417 × 10^3^/mm^3^ vs. 67 × 10^3^/mm^3^, *p* < 0.05). Leinoe et al. clarified that low P-selectin expression on stimulated AML platelets are due to reduced platelet synthesis as well as alpha-granules dysfunction [19]. Platelet abnormalities and/or dysfunction have also been reported in CML patients. However, this may be of particular importance in AML patients as AML is a rapidly progressive disease with severe quantitative platelet defect [20, 21].

In our study, both AML and CML patients had increased concentrations of F1 + 2 in relation to controls (*p* < 0.05). These results are similar to a study by Negaard et al. They found that the baseline values (before cancer therapy) of F1 + 2 were increased among of patients with hematological malignancies, including AML, compared to controls. Levels of F1 + 2 in plasma decreased significantly after cancer treatment (median 281.8 pM vs. 258.6 pM, *p* = 0.03), but remained significantly higher than controls (median 258.6 pM vs. 1.3 pM, *p* < 0.001) [22]. Thus, the state of hypercoagulability in myeloid malignancy is high at baseline and even after termination of cancer treatment. Moreover, the higher levels of F1 + 2 in AML than CML and controls are related to higher expression of tissue factors in AML [23, 24].

Moreover, sP-selectin was positively correlated to leukocyte count and F1 + 2 (*p* = 0.029 for both) only in AML patients, reflecting the interplay between platelet and/or endothelial activation and inflammation. This agrees with Wakefield and Myers who demonstrated that thrombosis and inflammation are inter-related [25]. Indeed, high leukocyte count in leukemia are associated with higher incidence of vascular complications. One potential mechanism to support this concept is that leukemic cells are able to activate endothelial cells via various cytokines, such as tumor necrosis factor alpha (TNF-α). Subsequent attachment of leukemic cells to the vessel wall via adhesion molecules induces coagulation activation and leukocyte aggregation [26]. This may explain the correlation between leukocyte count and sP-selectin as well as sP-selectin and F1 + 2 in AML patients of our study. Differently, in CML patients, plasma sP-selectin levels were not correlated with leukocyte count and F1 + 2. It might be possible that we could not detect direct correlation between those biomarkers because of the small group of CML patients.

On the other hand, PSGL-1 is predominantly expressed on myeloblast (AML blasts) rather than lymphoblast to interact with either P- or E-selectins. It is not only able to induce coagulation activation, but also promoting blast cells survival, drug resistance, and metastasis [8, 27]. Thus, inhibiting selectins interaction with their ligands may provide a new promising thromboprophylaxis, especially in AML in patients who carry high risk of bleeding during anticoagulant due to severe thrombocytopenia.

Some limitations of this study need to be acknowledged: (1) significant results may be hampered by a relatively small sample size; (2) we did not consider other congenital or acquired thrombophilia that might contribute to thrombotic risk. However, the results of this study may serve as a basis for future observational or interventional studies on thromboprophylaxis in hematological malignancies.

## Conclusions

In conclusion, this study provides direct evidence that AML and CML patients had an increased tendency to thrombosis. While CML patients had higher platelet and/or endothelial activation, hypercoagulable state are more pronounced in AML patients.

## Data Availability

The datasets used in the current study are available from the corresponding author on reasonable request.

## Abbreviations

AML: Acute myeloid leukemia
CAT: Cancer-associated thrombosis
CML: Chronic myeloid leukemia
ELISA: Enzyme-linked immunosorbent assay
F1 + 2: Prothrombin fragment 1 + 2
FAB: French-American British
Hb: Hemoglobin
IQR: Interquartile range
PSGL-1: P-selectin glycoprotein ligand 1
sP-selectin: soluble P-selectin
TAT: Thrombin-antithrombin complex
TF-MP: Tissue factor-bearing microparticles
TNF: Tumor necrosis factor
VTE: Venous thromboembolism
